# Interferon-β1a reduces plasma CD31+ endothelial microparticles (CD31+EMP) in multiple sclerosis

**DOI:** 10.1186/1742-2094-3-23

**Published:** 2006-09-04

**Authors:** William A Sheremata, Wenche Jy, Sylvia Delgado, Alireza Minagar, Jerry McLarty, Yeon Ahn

**Affiliations:** 1 Department of Neurology, Leonard Miller School of Medicine, University of Miami, Miami, Florida, USA; 2 Department of Medicine, Leonard Miller School of Medicine, University of Miami, Miami, Florida, USA; 3 Walter Coulter Laboratory, Leonard Miller School of Medicine, University of Miami, Miami, Florida, USA; 4 Department of Neurology, Louisiana State University Health Sciences Center, Shreveport, LA, USA; 5 Department of Medicine, Louisiana State University Health Sciences Center, Shreveport, LA, USA

## Abstract

**Background:**

A correlation between plasma CD31+ endothelial microparticles (CD31+EMP) levels and clinical, as well as brain MRI activity, in multiple sclerosis (MS) patients has been previously reported. However, the effect(s) of treatment with interferon-β1a (IFN-β1a) on plasma levels of CD31+EMP has not been assessed. In a prospective study, we measured plasma CD31+EMP levels in 30 patients with relapsing-remitting MS.

**Methods:**

Using flow cytometry, in a blinded study, we measured plasma CD31+EMP in 30 consecutive patients with relapsing-remitting MS (RRMS) prior to and 4, 12, 24 and 52 weeks after initiation of intramuscular therapy with interferon-β1a (IFN-β1a), 30 micrograms weekly. At each visit, clinical examination was performed and expanded disability status scale (EDSS) scores were assessed.

**Results:**

Plasma levels of CD31+EMP were significantly reduced from 24 through 52 weeks following initiation of treatment with IFN-β1a.

**Conclusion:**

Our data suggest that serial measurement of plasma CD31+EMP levels may be used as a surrogate marker of response to therapy with INF-β1a. In addition, the decline in plasma levels of CD31+EMP further supports the concept that IFN-β1a exerts stabilizing effect on the cerebral endothelial cells in pathogenesis of MS.

## Background

Multiple sclerosis (MS) is a uniquely human disorder of the central nervous system (CNS), which is characterized clinically by a relapsing course and neuro-pathologically by the presence of active inflammatory white and gray matter lesions in the brain and spinal cord [1]. Activated lymphocytes and macrophages are the main blood-borne cellular elements in the active inflammatory demyelination foci of the active MS plaques [1]. Endothelial adhesion and transendothelial migration of activated leukocytes through blood brain barrier (BBB) is thought to be a crucial step in formation of demyelinating lesions of MS within CNS [1-4].

Unlike other endothelial beds, cerebral endothelial cells have tight junctions which provide a highly impermeable anatomic and physiologic barrier to inward trafficking of various molecules and cells in the intravascular compartment.^2 ^Inflammatory cytokines such as tumor necrosis factor-α (TNF-α) and interferon-γ (IFN-γ) induce opening and redistribution of endothelial junctional proteins [2,3]. Increased permeability of the endothelial barrier of the BBB largely results from interactions among activated monocytes and T cells with cerebral endothelial cells, coupled with lymphokine and chemokine production, leading to cell adhesion to cerebrovascular endothelium and transendothelial migration across the BBB [2-4].

Upon activation by IFN-γ or TNF-α [5-7] and other cytokines released by activated lymphocytes and macrophages [3-7], endothelial cells release small membrane vesicles, known as endothelial microparticles (EMP). The released EMP carry adhesion molecules from the parent endothelial cells including P-selectin (CD62P), E-selectin (CD62E) [3,4], and platelet endothelial cell adhesion molecule (PECAM-1/CD31+) [4]. Although selectins are thought to be limited to an essential initial role in cell adhesion, i.e. slowing and initiating cell rolling, they have been shown to have a dominant role in allowing non-specifically activated cells to gain access to the CNS [5], independent from the integrin VLA-4 and VCAM-1 interaction.

Elevated plasma levels of plasma EMPs have been reported in MS, with values in stable patients in the normal range and elevations during exacerbations [11]. Since it has been suggested that the primary impact of interferon-β in MS is to reduce the permeability of the BBB [12], we prospectively, serially studied CD31+EMP in fresh plasma from 30 patients prior to and following initiation of interferon-β1a (IFN-β1a), 30 μg weekly (Avonex^®^). The effect of IFN-β1a on plasma levels of CD31+EMP, as a potential marker of response to treatment, was measured.

## Methods

### Patients

Thirty (30) relapsing MS patients who met the criteria of Poser et al [13] for clinically definite MS were serially studied over the course of 52 weeks. The study was approved by the institutional review board (IRB) and all patients provided signed informed consent. At least two neurologists concurred with the diagnosis of MS in all subjects. Patients were exacerbation-free for 3 months or more and stable for at least one month. No patient had received any immunosuppressive treatment and none had had any corticosteroids for at least 3 months prior to study entry. All patients had their expanded disability status scale (EDSS) scores determined prior to entry and at each examination at 4, 12, 24, and 52 weeks after initiation of treatment with IFN-β1a). An exacerbation of MS during the study was defined as a worsening of neurological impairment or the appearance of a new symptom(s) or abnormality attributable to multiple sclerosis, lasting 24 hours, and preceded by stability of at least one month.

Peripheral blood specimens were drawn prior to the first dose of IFN-β1a, and at 4, 12, 24, and 52 weeks.

### Control subjects

Blood specimens from 79 normal volunteers were studied concomitantly. All specimens from experimental subjects were coded and subsequent laboratory testing was performed blindly.

### Measuring EMP by flow cytometry

Venous blood was collected in citrate vacutainers using a 21 gauge needle. EMP assays were performed within four hours of blood collection to reduce nonspecific loss of EMP which we have found even when specimens are frozen at -70°C (unpublished). Blood was centrifuged at 160 × g for 10 min to prepare platelet rich plasma (PRP). The PRP was further centrifuged for 6 min at 1500 × g to obtain platelet-poor plasma (PPP). Then, a 25-μl aliquot of PPP was incubated with 4 μl of anti-CD42-FITC and 4 μl of anti-CD31-PE at ambient temperature for 20 min with gentle shaking (80 rpm). Following this, 0.5 ml of PBS was added. The EMP in the sample were measured using a Beckman Coulter EPICS XL flow cytometer. Detection of microparticles was triggered by FL2 (PE). Residual platelets were gated out by setting discriminator size < 1.0 μm. All microparticles positive for CD31 and negative for CD42 (CD31+/CD42-) were counted as EMP. The final concentration of EMP (count/μl) was calculated as previously described [11].

### Magnetic resonance imaging

Brain and spinal cord MRI were performed in all patients on a 1.5 T machine with a standard head coil prior to initiation of intramuscular treatment with IFN β1a (Avonex^®^), 30 μg weekly. The imaging protocol included sagittal T1-, axial T1-, T2-, and proton-density weighted images. All MRI scans were performed after infusion of gadolinium diethylenetriamine pentaacetic acid (Gd). Axial T1-weighted post-contrast and T2-weighted images were used for assessment of MS plaques. The images were independently interpreted by neuroradiologists blinded to the patients' clinical data. Whenever possible, follow-up mages were obtained every 24 weeks, in keeping with standard practice. Since none of the exacerbations were severe, no additional images were obtained at the time of exacerbations.

### Statistical analysis

Preliminary tests have shown that EMP exhibits a skewed, non-Gaussian distribution; therefore, nonparametric statistical tests were used in the analysis. *Friedman's nonparametric test for related *samples [18] was used to test for changes in EMP values from baseline during the subsequent weeks. This test is based on mean ranks of EMP at the various time points. Missing values reduced the number of patients with complete data at all time points, so multiple two-sample comparisons were also performed and should be interpreted with a lower type I error rate than usual. *Wilcoxon's paired nonparametric test *[19] was used to compare each time point to baseline. Repeated measures analysis of variance of the logarithm of EMP was used to test for a linear trend over all time points. Analysis was performed by SPSS statistical software (SPSS Inc., Chicago, Illinois) [20].

## Results

MS subjects had a mean age of 41.1 years at study entry (range18 to 60) and had a mean duration of illness of 5.4 years. All but one were women. Twenty six MS subjects completed the study. Two chose to stop treatment and attempt pregnancy prior to completion of the planned follow-up; one at 24 weeks and the other at 36 weeks. Each gave a blood sample for testing at that time. Both women successfully delivered healthy babies. Two other MS subjects moved out of Florida. No unexpected adverse experience was encountered as a result of IFN-β1a treatment. All patients in the MS and control groups were normotensive. A number of specimens were unsatisfactory and/or their results could not be retrieved for technical reasons. The mean age of 79 normal subjects was 42 and 80% of them were female. During the study MS patients were not treated with corticosteroids or any other immunosuppressive agents.

**Figure 1:**
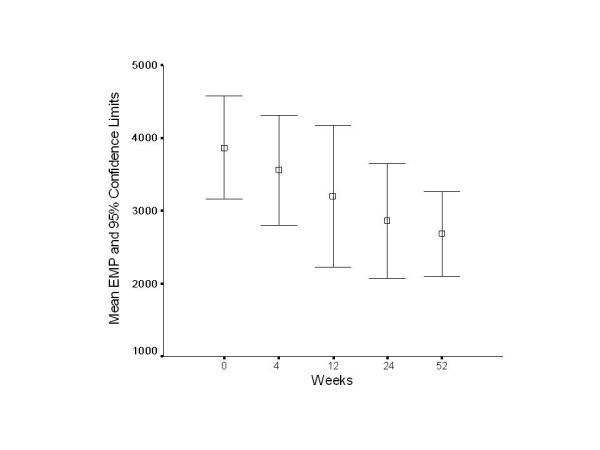
**EMP levels at different time points in IFN-β1a-treated MS patients**. EMP levels are significantly different over time, p < .016 as compared to baseline, by the Friedman nonparametric test. The levels decrease monotonically, and this trend is highly significant, p = 0.003. Error bars are 95% confidence limits of the mean. Note that the horizontal scale is not linear.

CD31+EMP: mean values in 79 normal subjects studied concomitantly with our serially studied multiple sclerosis patients was 697 ± 403/ml (mean ± standard deviation). Pretreatment mean CD31+EMP values for MS patients were 3866 ± 1900/ml (week 0) [Figure 1.]. After initiation of IFN-β1a treatment, CD31+EMP values were 3561 ± 1835/ml at week 4; 3203 ± 2193/ml at week 12; 2863 ± 1864/ml at week 24; and 2683 ± 1350/ml at week 52. IFN-β1a treatment was associated with a 29% reduction of plasma CD31+EMP levels at week 52. In the pretreatment group of MS patients, no values were within 1 SD of the mean for normals (1100) and only one of the values was within 2 SD. Of the CD31+EMP values obtained in the MS patients, 19 were within 2 SD of the mean for controls: 1 at baseline, 3 at week 4, 3 at week 12, 5 at week 24, and 7 at week 52. As shown in Figure 1, the measures at different time points are significantly different, p = 0.016. The trend in EMP monotonically decreases with time, as shown in figure 1. A test for linear trend was highly significant, p = 0.003.

**Figure 2:**
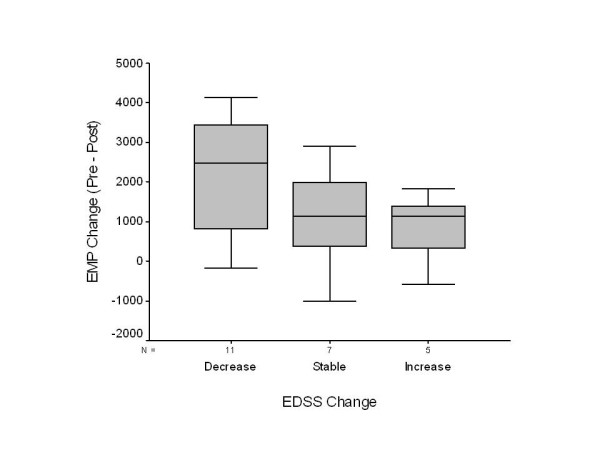
**Boxplot of changes in EMP levels with change in EDSS disability scores**. A change in EDSS score ≥ 1 is considered a decrease in disability and a change in score ≤ 1 is an increase in disability. Except for one case, the last EMP values were measured at 52 weeks.

Disability: At the end of the study, 11 of the subjects exhibited a one-grade or greater decrease in their EDSS scores; 7 did not change and 5 exhibited increases of their EDSS scores of one grade or more. Of the 11 with decreased EDSS, 10 exhibited decreases in plasma CD31+EMP by one SD of the normal or greater, and one did not. The values of plasma CD31+EMP in four MS subjects fell within 2 SD of the mean of the normals. The plasma CD31+EMP level in one MS subject, whose EDSS did not change, also fell within 2 SD of the normals. Of the five MS subjects with increased EDSS scores, plasma CD31+EMP values decreased in two and was within 2 SD of the normals in one. The relationship between EDSS score changes and EMP changes was not significant, p = 0.25, but, as shown in Figure 2, the changes were in the expected direction.

Exacerbations: Eight (8) relapses occurred in 6 patients while on IFN-β1a. Plasma CD31+EMP values increased in one who was tested at the time of a relapse. Values for the others were elevated, although two had initially decreased values at week 4, well prior to the time of relapse. Three patients exhibited evidence of onset of secondary progressive disease during the study. There was insufficient data for statistical analysis of these patients.

Gadolinium-enhanced brain MRI lesions were found in four of the 30 patients at entry to the study, and in two at the termination of the study. There was no correlation between the presence of the lesions, clinical symptoms and EMP values in 3 of the patients, but one did have an increase in EDSS and an increase in plasma CD31+EMP level. There was insufficient data for statistical analysis of these findings.

## Discussion

We have observed a decrease in plasma CD31+EMP following initiation of intramuscular treatment of MS patients with IFN-β1a, 30 μg weekly. This decrease reached significance at week 12 and became more significant from week 24 to the termination of the study. At study entry only one (3%) of all pretreatment CD31+EMP values in MS patients were within normal range (≤ 2 SD of mean normal values), although all of the MS patients were clinically stable and only 4 of 30 exhibited abnormal brain MRIs with contrast enhancing lesions. In contrast, 29% of EMP values were within this range at 52 weeks.

The decrease in plasma CD31+EMP levels with IFN-β1a treatment undoubtedly reflects a reduction in CD4+ cell interaction with the endothelium and transendothelial migration of activated leukocytes across the blood brain barrier (BBB). Although this could not be established with the current study design, such decrease likely correlates with the reestablishment of the integrity of the BBB [10]. More frequent serial brain MRI studies may have strengthened this conclusion. These elevated plasma levels of CD31+EMP in untreated MS patients who clinically appear to be stable suggests the presence of continuing low-level damage to the BBB, at least to the endothelial component of the BBB [10].

Several lines of evidence support the essential role of the breaching of the BBB in the development of disease in experimental allergic encephalomyelitis (EAE) [4,8,9,14] as well as in MS [2,10,15,16]. In recent clinical trials with natalizumab, it has been consistently shown that blocking adhesion molecules is dramatically beneficial in MS [15,16]. This humanized monoclonal antibody against the α4β 1 integrin (VLA-4, CD106), which is expressed on activated lymphocytes and monocytes, prevents their binding to endothelial cells and egress into brain and spinal cord [14]. As a result it dramatically reduces the number of new lesions visualized by MRI, as well as clinical relapses, and improves the well-being of MS patients [15,16]. These results further confirm the central role of cell adhesion and transendothelial migration of lymphocytes and monocytes in the pathogenesis of MS.

To further support the role of cerebral endothelium interactions with activated leukocytes in pathogenesis of MS and the stabilizing effect of β-interferons on the endothelial barrier, Jimenez et al [21] used an *in vitro *model of monocyte migration through cerebral endothelial cell monolayers to demonstrate that monocytes form complexes with CD31+EMP (monocyte:CD31+EMP complexes) and that this, in turn, facilitates transendothelial migration of monocytes. These investigators further showed that addition of IFN-β1b to this *in vitro *model inhibits the formation and transendothelial migration of the monocyte:CD31+EMP complexes. Other investigators have also demonstrated that β-interferons counteract the effects of pro-inflammatory cytokines on the integrity of the endothelial layer of the BBB and, therefore, stabilize endothelial integrity [22,23]. Other possible explanations for the observed effect of IFN-β1a on plasma levels of CD31+EMP involve the protective effects of β-interferons as a class on the endothelial layer of the BBB. These protective effects include increased expression of occludin [24], decreased production of matrix metalloproteinases and increased levels of tissue inhibitor of matrix metalloproteinases [25], and counteraction of the disintegrating effects of IFN-γ on endothelial tight junctions and barrier function [24]. The results of the present study, together with the findings of other investigators, suggest that IFN-β1a, through its stabilizing effects on the cerebral endothelial cells, decreases the number of released CD31+EMP and the pre- and post-treatment plasma levels of CD31+EMP, and that these levels may serve as a surrogate measure of disease activity in MS and patients' response to therapy. Further serial studies with frequent brain MRI and more frequent CD31+EMP assays will be needed to support the utility of such studies.

## Competing interests

The author(s) declare they have no competing interests.

## Authors' contributions

WAS, SD, AM designed and performed the study, examined the patients, and drafted the manuscript. WJ and YA performed the experiments described. JM analyzed the data and prepared the figures. All authors read and approved the final manuscript.
